# Does group-based trajectory modeling estimate spurious trajectories?

**DOI:** 10.1186/s12874-022-01622-9

**Published:** 2022-07-14

**Authors:** Miceline Mésidor, Marie-Claude Rousseau, Jennifer O’Loughlin, Marie-Pierre Sylvestre

**Affiliations:** 1 Centre de Recherche du Centre Hospitalier de l’Université de Montréal (CRCHUM), Université de Montréal, Montréal, QC Canada; 2 Department of Social and Preventive Medicine, Université de Montréal, Montréal, QC Canada; 3 Centre Armand Frappier Santé Biotechnologie, Institut National de La Recherche Scientifique, Laval, QC Canada

**Keywords:** Group-based trajectory modeling, Simulated subgroups, Average posterior probability, Relative entropy, Mismatch

## Abstract

**Background:**

Group-based trajectory modelling (GBTM) is increasingly used to identify subgroups of individuals with similar patterns. In this paper, we use simulated and real-life data to illustrate that GBTM is susceptible to generating spurious findings in some circumstances.

**Methods:**

Six plausible scenarios, two of which mimicked published analyses, were simulated. Models with 1 to 10 trajectory subgroups were estimated and the model that minimized the Bayes criterion was selected. For each scenario, we assessed whether the method identified the correct number of trajectories, the correct shapes of the trajectories, and the mean number of participants of each trajectory subgroup. The performance of the average posterior probabilities, relative entropy and mismatch criteria to assess classification adequacy were compared.

**Results:**

Among the six scenarios, the correct number of trajectories was identified in two, the correct shapes in four and the mean number of participants of each trajectory subgroup in only one. Relative entropy and mismatch outperformed the average posterior probability in detecting spurious trajectories.

**Conclusion:**

Researchers should be aware that GBTM can generate spurious findings, especially when the average posterior probability is used as the sole criterion to evaluate model fit. Several model adequacy criteria should be used to assess classification adequacy.

**Supplementary Information:**

The online version contains supplementary material available at 10.1186/s12874-022-01622-9.

## Background

The evolution of health outcomes is often heterogenous across individuals, prompting researchers to use methods to identify subgroups of individuals with similar patterns over time [1]. Trajectory modelling is used to identify subgroups with the aim of predicting future outcomes or targeting an intervention based on the patterns observed [1]. Examples of behaviors and health outcomes include trajectories of cigarette smoking [2] and progression of disability among patients with multiple sclerosis [3, 4].

Group-based trajectory modeling (GBTM) is one of the most frequently used approaches to identify subgroups of people in longitudinal data. The popularity of GBTM is in part due to the availability of routines in standard statistical programs (e.g., PROC TRAJ in SAS, or TRAJ in Stata) that provide easy-to-interpret visual summaries of the data. In addition, GBTM is less computationally demanding, simpler to fit and easier to use in samples with smaller numbers of observations than its most popular alternative, the latent growth mixture modeling (LGMM) approach [5, 6]. Such attractive properties, however, are mitigated by making strong assumptions about the distribution of trajectories. For example, while LGMM estimates within-subgroup variance parameters, these parameters are set to zero in GBTM, which eases convergence of the model, but makes the strong assumption that individual trajectories are homogeneous within subgroups [1].

Studies have suggested that violations of GBTM assumptions may create spurious findings such as the identification of trajectory subgroups that are statistical artefacts rather than homogeneous subgroups in the data [7–9]. Misspecification of the distribution of the outcome may also lead to identifying spurious subgroups [8, 10]. For example, estimating a GBTM using a continuous variable with a non-normal distribution tends to inflate the number of subgroups because GBTM relies on a mixture of normal distributions to estimate the non-normal distribution of the variable [8, 10]. Studies have also shown that models that do not estimate within-class variability may induce bias, in addition to reducing subgroup recovery and accuracy of the classification [11, 12]. Further, Vachon et al. recently coined the term “rainbow effect” [9] to describe the set of parallel trajectories observed in many GBTM applications [3, 13, 14]. The rainbow effect appears when the distribution of the values of the variable under study does not correspond to a mixture of several homogeneous trajectory subgroups but rather to gradations on a continuum of values [9].

While criteria of model adequacy for GBTM exist, it is unclear whether they can guard against the identification of spurious findings. The Guidelines for Reporting on Latent Trajectory Studies (GRoLTS) recommend using the Bayesian Information Criteria (BIC) to select the number of subgroups and the order of the polynomial terms used to model the shapes of trajectories [5, 15]. Criteria such as the average posterior probability (APP) and relative entropy are then used to assess the adequacy of the classification of individual trajectories in the subgroups identified using GBTM [1, 5].

In this paper, we describe a case-study using simulated data to illustrate that: (i) GBTM can produce spurious findings; and (ii) model adequacy criteria can fail to identify model misspecification. Despite guidelines [5] and best practices [16] recommending using numerous criteria to select the number of trajectory subgroups, recent reviews of trajectory modelling using GBTM in the health-related literature [17–20] suggest that BIC and APP are often the only criteria used. Herein we investigate the BIC and the APP because they are currently the only criteria for selecting the number of trajectory subgroups and model adequacy, respectively that are computed by the software used by most researchers to estimate GBTM [21, 22]. We also consider relative entropy and mismatch as additional criteria because they can easily be computed in any software including SAS (see Appendix for the codes). We generated data similar to applications published in the health-related literature with a focus on scenarios investigating the variability of the variable under study within and across trajectory subgroups over time. We then used real-life data to support the results of the simulations.

## Methods

Before presenting the design of the simulation study, we briefly present GBTM and describe the conventional modelling strategy used to select the number of subgroups and the shapes of trajectories, in addition to the model adequacy criteria used to assess classification adequacy.

### Group-based trajectory modeling [1]

Let Y_i_ = {y_i1_, y_i2_, …, y_it_) be a sequence of $t$ measurements of a normally distributed variable Y for $i$=1, …, N participants. GBTM is a finite mixture model for which the following equation describes the likelihood of a participant’s observed repeated measures:

$$P\left({Y}_i\right)={\sum}_{j=1}^j{\pi}_j{P}^j\left({Y}_i\right)$$

in which ${{P}^{j}(Y}_{i})$ represents the conditional probability of ${Y}_{i}$ given membership in group *j* and ${\pi }_{j}$ is the mixture parameter that captures the probability that a randomly selected participant belongs to subgroup *j*. The model is estimated using a latent class formulation in which each subgroup *j* has a specific sets of regression coefficients that corresponds to the variables indexing time, for example $:$

1 $${{y}_{it}^{*j}=\beta }_{0}^{j}+ {\beta }_{1}^{j}{Time}_{it}+ {\beta }_{2}^{j}{Time}_{it}^{2} + {\beta }_{3}^{j}{Time}_{it}^{3}+ {\varepsilon }_{it}$$

$${\varepsilon }_{it}\sim N\left(0, \sigma \right)$$

$j$ indicates trajectory subgroups

${y}_{it}^{*j}$ is a latent variable which makes the link between the variable under study and the time variables. In Eq. (1), temporal variations in Y are captured using cubic polynomials, but models with different polynomial orders can be used. Each trajectory subgroup can have a different shape that is determined by the coefficients ${\beta }_{0}^{j}, {\beta }_{1}^{j},{ \beta }_{2}^{j}, {\beta }_{3}^{j}$ assigned to the time polynomials in Eq. 1. In this paper, we focus on the censored normal distribution but the model can be adapted for variables that follow a Bernoulli or Poisson distribution [1].

Individual posterior probabilities of subgroup membership can be derived from estimates of the mixing parameter ${\pi }_{j}$, in addition to estimated regression coefficients from Eq. 1. Such posterior probabilities can be used to assign individuals to the subgroup for which their probability is the highest, according to the maximum-probability assignment rule [1, 23].

### Model selection and assessment

In most GBTM applications [5], the optimal number of trajectory subgroups is selected by estimating models with an increasing number of trajectories, using the BIC approximation to the Bayes factor as long as the difference between two adjacent models (e.g., 2 and 3 subgroups) was greater than 10 [5]. Cubic polynomials are generally used to represent the trajectories. Once the number of trajectories is selected, the model is usually refitted using lower-order terms if the higher-order terms are not statistically significant at 5% [1].

Model adequacy may then be assessed using several criteria. The APP is calculated for each trajectory subgroup by averaging over the individual posterior probabilities of trajectory membership of individuals assigned to the subgroup. APPs greater than 70% across trajectory subgroups are considered indicative of adequate classification [1]. The relative entropy measures the degree of classification accuracy of placing participants into a trajectory based on their posterior probabilities. A value greater than 0.80 indicates less classification uncertainty [24, 25]. Finally, mismatch is the difference between the estimated probability of subgroup membership and the proportion of individuals classified in that subgroup based on the highest posterior probability. The correspondence between these two probabilities decreases as assignment error increases [1]. Therefore, a difference close to 0 suggests an adequate fit [1, 16].

### Data simulation and scenarios

**Fig. 1:**
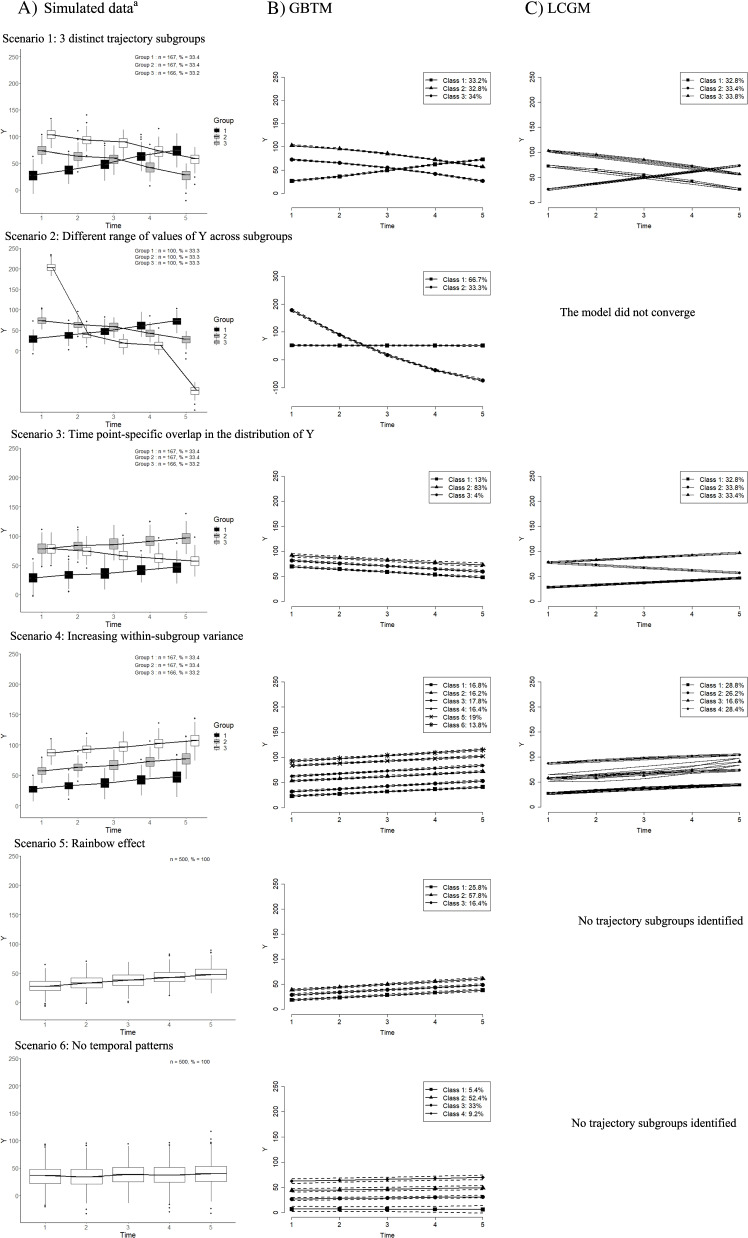
Simulated data (left panel) and identified trajectories (right panel) for each scenario ^a^To make the boxplots for scenario 1–4 more legible, the boxes representing each subgroup at each time point were shifted slightly so that they would not overlap

We simulated six plausible scenarios illustrating problems that may arise in practice, two of which mimicked published analyses [9, 26]. Rather than using a simulation study with many iterations, we opted for simulated case studies to analyze each scenario in-depth by contrasting the estimated results with the true data generating mechanisms. We simulated longitudinal data for i = 1, …, n = 500 individuals with t = 5 repeated measures of the variable ${Y}_{it}$, except for scenario 2 in which we considered a smaller dataset (*n* = 300). The left panel of Fig. 1 shows subgroup-specific box plots that represents the distribution of the simulated data for each scenario and the relative size of each subgroup. The simulation model is described in Appendix 1, while the scenario-specific parameters used in the simulation are presented in Table S1. GBTM assumes that the residual variance is constant over time and similar across subgroups. To account for non-homogeneity between subgroups [25], we used a more flexible data generating process. Such heterogeneity is taken into account in linear mixed models through the random effects. Scenarios 1 to 4 represent examples in which ${Y}_{it}$ was simulated according to three subsets of trajectories, while Scenarios 5 and 6 illustrated situations in which there were no subgroups in the simulated data.

Scenario 1 is a benchmark example in which GBTM is expected to capture the correct number of subgroups and shapes of trajectories because the three simulated trajectory subgroups were clearly distinct. The first trajectory represents a rapidly increasing pattern, while the other two represent parallel decreasing patterns. Scenario 2 represents a dataset in which the range of values of *Y* differs importantly across subgroups. To this end, we modified Scenario 1 to include a steeper slope of the increasing trajectory, with a range of values twice as large as in Scenario 1. Scenario 3 investigated the impact of overlap in the subgroup-specific distributions of *Y* at given time points during follow-up. We simulated two subgroups with increasing parallel trajectories and a subgroup with a decreasing trajectory. The distribution of *Y* in the two increasing trajectories largely overlapped at time 1, while the decreasing trajectory overlapped with the first increasing trajectory at time 5. Scenario 4 mimicked a study of cigarette smoking initiation [26] in which time zero represents the onset of cigarette smoking. Participants all started at time zero and cigarette consumption evolved according to three initially distinct trajectories in which the distributions overlapped towards the end of follow-up due to increasing within-subgroup variability. This scenario would be expected to cause GBTM to yield spurious trajectories due to the high subgroup variability. Scenario 5 illustrated the rainbow effect coined by Vachon et al. to describe a variable with a distribution on a continuum rather than distinct trajectories [9]. We generated a uniform continuum of individual trajectories centered around an increasing trend. Finally, to assess whether GBTM identified distinct trajectories in the absence of any subgroups [7, 27], we simulated a scenario in which there were no subgroups of homogeneous trajectories (Scenario 6).

### Data analysis

For each scenario, we estimated models with a cubic term and considered 1 to 10 trajectory subgroups. We used the Bayes factor to select the number of subgroups and removed higher order terms that were not statistically significant at 5%. Model adequacy criteria were computed for the models selected.

We compared the estimated number of trajectories and the relative size of each subgroup with that generated in the simulations. We also compared the estimated trajectory shapes with those generated using visual inspection.

To better understand the potential limitations of GBTM, trajectory subgroups were also identified in sensitivity analyses using a latent class growth models (LCGM) which estimate within-class variability. LCGM were estimated using the *hlme* function of the *latent class mixed models* (*lcmm*) package in R software (https://cran.r-project.org/web/packages/lcmm/lcmm.pdf). The models include a subgroup-specific random intercept that allows for the estimation of different variances within trajectory subgroups [28]. We contrasted the number and shapes of trajectories obtained across GBTM and LCGM.

Simulations and analyses were conducted in SAS (version 9.4, SAS Institute Inc., Cary, North Carolina, USA) using PROC TRAJ (https://www.andrew.cmu.edu/user/bjones/) to estimate GBTM. Trajectories were plotted using R.4.0 (R Core Team, Vienna, Austria; 2014).

## Results

Figure 1 presents simulated data for each scenario in the left panel, and trajectories estimated using GBTM and LCGM, respectively in the right panel. Figure 1 also includes the relative size of each generated and estimated subgroup. Model adequacy criteria including APP, entropy and mismatch are reported in Table 1 for GBTM.

**Table 1:** Model adequacy criteria using GBTM for each scenario

Criteria^a^	Scenario 1: Three distinct trajectory subgroups							Validity of classification
		1	2	3				
Average posterior probability		1.00	0.99	0.98				All criteria suggest good classification
Mismatch		0.03	0.23	-0.26				
Relative entropy	0.98							
	Scenario 2: Different range of values of Y values across subgroups							
Average posterior probability		1.00	1.00					All criteria suggest good classification
Mismatch		0.00004	-0.00004					
Relative entropy	1.00							
	Scenario 3: Time point-specific overlap in the distribution of Y							
Average posterior probability		0.93	**0.56**	0.92				All criteria suggest that the classification is not optimal
Mismatch		**26.44**	**-35.27**	**8.82**				
Relative entropy	**0.34**							
	Scenario 4: Increasing subgroup with variance							
		1	2	3	4	5	6	
Average posterior probability		0.91	0.89	0.90	0.86	0.91	0.88	All criteria suggest good classification
Mismatch		0.19	0.05	0.23	-0.89	0.49	-0.07	
Relative entropy	0.87							
	Scenario 5: Rainbow effect							
		1	2	3				
Average posterior probability		0.84	0.83	0.84				Mismatch and entropy suggest that the classification is not optimal
Mismatch		**1.30**	**-2.89**	**1.59**				
Relative entropy	**0.65**							
	Scenario 6: No temporal patterns							
		1	2	3	4			
Average posterior probability		0.89	0.89	0.84	0.90			Mismatch and entropy suggest that the classification is not optimal
Mismatch		0.52	**-1.97**	0.45	**1.00**			
Relative entropy	**0.66**							

^a^ APP > 0.70 and mismatch close to 0 suggest that the classification is good. Entropy close to 1 indicates that participants were classified with more confidence. Bold values indicate poor classification

*Scenario 1: three distinct trajectory subgroups*

In the benchmark scenario, GBTM recovered the correct number and shape of trajectories and the relative size of the subgroups. All three criteria suggested adequate classification. LCGM recovered the correct number and shapes of the trajectory subgroups.

*Scenario 2: different range of values of Y values across subgroups*

GBTM identified two trajectory subgroups, whereas three were simulated. The shape and relative size of the steep trajectory subgroup was well-identified, but the increasing and decreasing simulated trajectories were combined into one single flat trajectory that included 66% of participants. All three criteria suggested adequate classification. We estimated LCGM with different numbers of trajectory subgroups, but none converged.

*Scenario 3: time point-specific overlap in the distribution of Y*

The relative size of the subgroups and the shapes of the trajectories did not match those simulated. The simulation included two increasing trajectories and one decreasing trajectory; GBTM identified three parallel decreasing trajectories. While the mismatch was high and the relative entropy was poor, suggesting poor classification, the APP suggested poor classification for one trajectory subgroup only. LCGM recovered the correct number and shape of the trajectory subgroups.

*Scenario 4: increasing within-subgroup variance*

Instead of the 3 trajectories that were simulated, GBTM identified 6 trajectories by dividing each of the 3 simulated subgroups into two subgroups with parallel trajectories. The shape of estimated trajectories corresponded to those generated. All three criteria suggested adequate classification. LCGM failed to identify the correct number of trajectories (i.e., four trajectory subgroups were identified, although three were simulated).

*Scenario 5: rainbow effect*

Although data were generated as a uniform continuum of individual trajectories, GBTM identified three subgroups, with relative sizes including 25.8%, 57.8% and 16.4% of the sample. The APP suggested satisfactory classification for each subgroup. Unlike the APP, the relative entropy and mismatch identified misclassification. LCGM did not identify any trajectory subgroups for this scenario.

*Scenario 6: no temporal patterns*

Although no patterns or subgroups were generated, GBTM identified four trajectories with relative sizes of 5.4%, 52.4%, 33% and 9.2%, suggesting that GBTM identified random fluctuations as trajectory subgroups. The APP suggested adequate classification. The mismatch indicated poor classification for two of the four trajectory subgroups. The relative entropy indicated poor classification. Again, LCGM did not identify any trajectory subgroups.

There was agreement among the three criteria for classification adequacy for three of the six scenarios. This included Scenario 1 for which GBTM correctly identified the number and shapes of trajectories and the three criteria suggested adequate classification, but also Scenarios 2 and 4 in which all three criteria failed to identify the spurious trajectories. In the remaining three scenarios, both the relative entropy and the mismatch criteria correctly suggested poor classification, while the APP only identified it once.

Overall, LCGM outperformed GBTM in the presence of time point-specific overlap in the distribution of Y (Scenario 3) and when there were no trajectory subgroups in the data (Scenarios 5–6).

## Application

Data from the Nicotine Dependence in Teens (NDIT) Study were used to identify trajectories of difficulty initiating and maintaining sleep. The NDIT study is a longitudinal investigation of 1293 students recruited in 10 Montreal high schools in 1999–2000 [29]. Self-report questionnaires were completed by students every 3 months from grade 7 to 11 (i.e., total of 20 data collection cycles during high school). The study was approved by the Montreal Department of Public Health Ethics Review Committee, the McGill University Faculty of Medicine Institutional Review Board, and the Ethics Research Committee of the Centre de Recherche du Centre Hospitalier de l’Université de Montréal. Difficulty initiating and maintaining sleep was assessed by: “During the past 3 months how often have you had trouble going to or staying asleep?” Response options included “never”, “rarely”, “sometimes”, or “often”. We restricted the analysis to students from the largest school to avoid modelling the within-school correlation induced by the clustered sampling. The illustrative example is thus estimated using 169 students with at least three observations during the follow-up.

We considered models with 1–10 trajectory subgroups and assumed a censored-normal distribution for difficulty initiating and maintaining sleep, which is used routinely in applications of GBTM to ordinal variables [30] when other options available (i.e. binomial, zero-inflated Poisson) are not suitable. For example, Jones and Nagin used a censored-normal distribution to study the level of childhood opposition, which varied from zero to seven [30]. Comparison of the Bayes factor across models resulted in selecting the three-subgroup model (Table S5). While the APP and the relative entropy for the model suggested adequate classification (Table S6), the absolute value of the mismatch criterion was larger than two for two trajectory subgroups, suggesting poor classification.

**Fig. 2:**
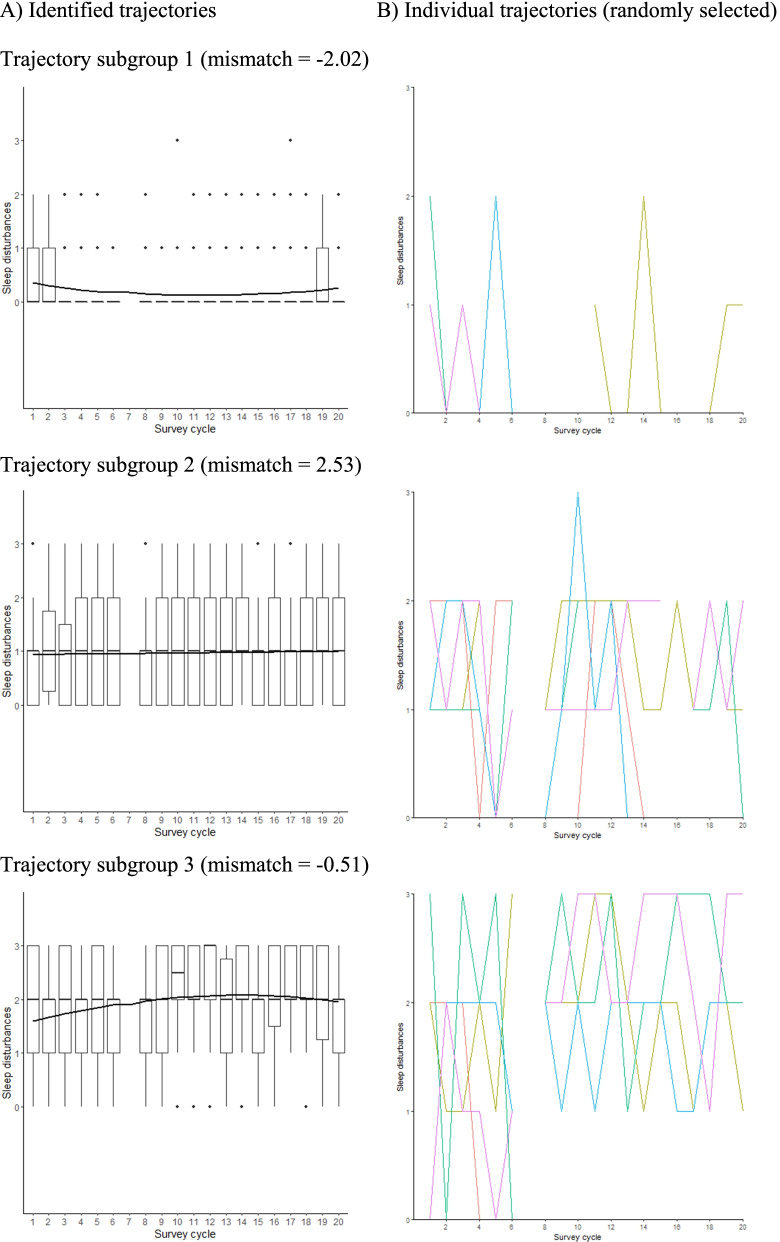
Individual trajectories of difficulty initiating and maintaining sleep for identified trajectories (left panel) and a sample of participants (right panel), NDIT Survey cycles 1–20

We investigated the source of disagreement between the classification criteria by inspecting the distribution of difficulty initiating and maintaining sleep and the magnitude of individual changes in each of the trajectory subgroup. The left panel of Fig. 2 shows estimated trajectories superimposed on subgroup-specific box plots of difficulty initiating and maintaining sleep, while the right panel displays random selection of individual patterns in each subgroup. Estimated trajectories were mostly flat and mirrored the subgroup-specific median difficulty initiating and maintaining sleep. The boxplots suggest larger cycle-specific variation in subgroups 2 and 3 relative to subgroup 1. The right panels revealed important within-individual acute changes in difficulty initiating and maintaining sleep that were not reflected in the estimated trajectories because they occurred throughout the follow-up and not at a specific age for a significant subset of the sample.

Based on the results of our simulation, GBTM may provide the misleading impression that individual patterns of difficulty initiating and maintaining sleep are flat through adolescence. That only the mismatch criterion suggested poor classification may reflect that by assessing the discordance between estimated and assigned group probabilities, the mismatch criterion provides a more general fit assessment than the other two criteria.

## Discussion

We used simulated and real-life data to demonstrate how GBTM may create spurious trajectories in scenarios that mimic published studies. We further showed that the widely used APP criteria for classification adequacy failed to detect spurious trajectories in most scenarios.

We found that GBTM creates spurious trajectories when there are no trajectories in the data, an observation that others have reported previously [7, 8]. Aligned with Vachon et al. [9], we also observed that GBTM identified spurious subgroups when individual trajectories are on a single continuum of values of the variable under study. It thus appears that GBTM overestimated the number of trajectories when individual trajectories have the same shape and are distributed on a continuum around the mean trajectory. This result suggests that the assumption of within-subgroup homogeneity may be the main driver behind the spurious identification of subgroups in the “rainbow effect” scenario, since LCGM did not identify trajectory subgroups.

Our results further suggest that GBTM overestimated the number of subgroups in several scenarios of simulated data which included subgroups. For example, in the scenario in which within-subgroup variance increased over time, GBTM identified twice as many subgroups as expected, although it did identify the correct trajectory shapes. This suggests that the large variance observed towards the end of follow-up drove the model to split each trajectory subgroup into two. While this may not be as problematic as not identifying a subgroup or misspecifying the shape of a trajectory, doubling the number of trajectories necessarily results in smaller subgroups. This in turn may complicate inference in terms of detecting associations between the trajectories and factors or distant outcomes by unduly increasing the degrees of freedom and limiting the precision of the estimates.

Finally, spurious trajectories were observed in scenarios with different ranges of values of the variable under study across subgroups or when time-point specific of the variable under study overlapped in the distribution of the variable under study. These two scenarios were characterized by high within- and between-subgroup variability, which may have affected the performance of GBTM. Moreover, the assumption that individual trajectories are homogeneous within subgroups may not be valid in these two scenarios. Hence, LCGM may be more appropriate in terms of taking variability between subgroups into account. However, LCGM are known to have convergence issues [11, 31]; they estimate a larger number of parameters than GBTM and are thus more computationally intensive. This was observed in our simulation study in which LCGM failed to converge in the scenario that had the largest within-subgroup variation (Scenario 2).

Our results emphasize the importance of using more than one criterion to assess classification adequacy, as others have suggested previously [16, 32]. While the GRoLTS checklist prioritizes measures of relative entropy to assess classification adequacy [5], a large number of applications relied on the APP. For example, a recent literature review on cigarette smoking trajectories in youth [2] showed that, among 17 studies which reported model adequacy criteria, 15 (88.2%) used the APP and two (11.8%) used entropy. The high level of reporting APP may relate to its availability in PROC TRAJ, which was used in most studies reviewed. We provide the SAS code for the relative entropy (inspired by Blaze’s work [33] and the mismatch calculation in the Appendix (Code S1). Cut-offs to assess the accuracy of the classification have been proposed for some criteria (e.g., APP, relative entropy). However, our results suggest that a criterion above the cut-off should not be considered sufficient.

The finding that all three model adequacy criteria failed to identify spurious trajectories in two of the six scenarios also highlights the importance of considering additional methods to assess the validity of findings. Our results do not suggest that LCGM always performs better than GBTM and should therefore always be used. Further, they do not suggest that it is possible, a priori, to determine the correct model to use. Therefore, aligned with Sijbrandij et al. [11], we suggest that researchers estimate more than one latent class model and choose the optimal model based on different fit indices as well as interpretation of the subgroups. Our other recommendations include: (i) a visual inspection of individual patterns within subgroups using a spaghetti plot (i.e., simultaneously in small samples or in a random subset of observations in larger samples); (ii) plotting raw data by subgroup as estimated by GBTM or using a discriminating time-invariant characteristic such as sex/gender or socio-economic status to investigate whether individual trajectories are homogeneous across subgroups; (iii) discussion with content experts; and (iv) comparing GBTM results to alternative clustering algorithms or latent class models such as the 3-step procedure proposed by Leffondré et al. [34, 35] and the latent class linear mixed model [28]. However, the partial implementation of such models in the most popular statistical software may limit the feasibility of the latter point.

When possible, substantive criteria may also help increase confidence in the validity of the subgroups identified. For example, Vachon et al. [9] used four criteria to investigate whether the development of alcohol use is continuous or categorical: i) trajectory analyses indicating a greater number of subgroups always fit the data better than a lesser number; ii) a relatively normal distribution of participants across trajectory arcs; iii) relatively parallel subgroups; and iv) changes in external correlates of trajectories reflecting rank-order stability of the trajectories. The authors concluded that their results support the notion that the development of alcohol use is continuous, and the existence of true trajectories can only be defined by strong, discriminating, and categorical factors that place participants on a deterministic natural course [9]. This argument was reinforced by Smeden et al., who argued that a cluster-based approach must build on existing theory to support the identification of valid subgroups [36].

Our work has clinical and substantive implications. Systematic reviews of trajectory analyses in health-related research [1–4, 20] report heterogeneous results, suggesting that studies generally do not replicate trajectories previously identified. This may be explained partially by spurious findings [20], although differences across samples including number of data points and longer time intervals between data points may also underpin failure to replicate previous findings. The use of GBTM in studies linking patterns of exposure to outcome measures at the end of follow-up (or subsequent clinical outcomes) may also lead to invalid results if the trajectories identified are spurious. Further, the use of trajectory analysis may not optimally relate patterns to outcomes. Sylvestre et al. show that trajectories are not always the most informative representation of longitudinal data because they can sometimes refer to different points in time during follow-up or to different subgroups, making it necessary to define specific periods over which to compare individuals [18]. In addition, use of trajectory subgroups may result in loss of power and in this case, using discrete measures may be more informative. Moreover, trajectory subgroups modeled as an exposure variable necessitates defining a reference subgroup, which may render comparison with other studies challenging.

Limitations of our work include that our investigation was restricted to BIC to select the number of trajectory subgroups, and three commonly used model adequacy criteria. Other criteria such as the Lo-Mendall-Rubin Likelihood Ratio (LMR-LRT) and the parametric bootstrapped likelihood ratio test (BLRT) sometimes outperform the BIC in selecting the number of groups [15, 37], but they are not yet computed in PROC TRAJ. Elbow plots of BIC values can also help in the selection of the number of subgroups [38]. Although sample size cutoffs are always used in the literature, we did not report this in the paper because all selected models had sample sizes greater than 5 (see Appendix, Tables S2-S3). While several alternative criteria exist to assess model adequacy, we excluded those that did not contribute any additional information over the criteria under investigation. For example, the odds of correct classification, which represents the ratio of the odds of a correct classification into each subgroup based on the maximum probability classification rule and the estimated class membership proportions, is a reformulation of the APP [1, 16]. However, recently proposed criteria such as the discrimination index should be evaluated [32]. Our analytical plan involved identifying the number of subgroups using the Bayes criterion and may not reflect the iterative process that is used in empirical studies in which classification criteria or substantive knowledge may lead to the selection of a different number of subgroups [5]. Nevertheless, our results suggest that a careful use of assessment criteria is warranted. Simulation studies involving a large number of iterations are required to quantify the bias caused by improper use of GBTM, although this will require automating all the modelling steps involved in fitting GBTM, which may not reflect the decision-making process that analysts use.

This paper extends previous works illustrating how improper assessments of model adequacy and variance constraints [11] in GBTM may lead to spurious findings. For example, insufficient flexibility in the modelling of nonlinear trajectories (i.e., using polynomials of orders that are too low) may lead to the so-called “cat’s cradle effect”, a tendency of GBTM to repeatedly identify four trajectories across different datasets (i.e., including both low and high flat patterns, in addition to increasing and decreasing trajectories crossing towards the middle of follow-up [39–41]). Analysts need to be aware of such pitfalls and the recent guidelines on the conduct and reporting of trajectory studies are a welcome addition to the growing literature on GBTM [5, 16].

In conclusion, while GBTM may provide a useful depiction of longitudinal data, it should be used with caution. Due to the data driven nature of the method, the accuracy of the results should be assessed and reported at each step of the analysis.

## Supplementary Information

**Additional file 1: Appendix 1. **Simulation model. **Table S1.** Parameter values used in the simulation for each scenario. **Table S2.** Fit indices for the trajectory model using group-based trajectory modeling, by number of trajectory subgroup for scenarios 1-3. **Table S3.** Fit indices for the trajectory model using group-based trajectory modelling, by number of trajectory subgroup for scenarios 4-6. **Table S4.** Bayesian Information Criteria for the trajectory model using latent class growth model, by number of trajectory subgroup. **Table S5.** Fit indices for the trajectory model for difficulty initiating and maintaining sleep, by number of trajectory subgroup. **Table S6.** Model adequacy criteria for the trajectory model for difficulty initiating and maintaining sleep. **Code S1. **Example of SAS code for the calculation of entropy and mismatch.

## Data Availability

All data generated and analyzed for simulations during this study are included in this published article [Appendix, Table S1]. The NDIT dataset used and analyzed during the current study are available from the corresponding author on reasonable request.

## Abbreviations

APP: Average posterior probability
BIC: Bayesian information criteria
GBTM: Group-based trajectory modeling
GRoLTS: Guidelines for Reporting on Latent Trajectory Studies
LGMM: Latent growth mixture modeling
NDIT: Nicotine Dependence in Teens
