# Supplementary material for: Does group-based trajectory modeling estimate spurious trajectories?
Source: BMC Med Res Methodol. 2022 Jul 14;22:194. doi: 10.1186/s12874-022-01622-9 (PMC9281109; doi:10.1186/s12874-022-01622-9)
Supplement: Supplementary file 1 — Additional file 1: Appendix 1. Simulation model. Table S1. Parameter values used in the simulation for each scenario. Table S2. Fit indices for the trajectory model using group-based trajectory modeling, by number of trajectory subgroup for scenarios 1-3. Table S3. Fit indices for the trajectory model using group-based trajectory modelling, by number of trajectory subgroup for scenarios 4-6. Table S4. Bayesian Information Criteria for the trajectory model using latent class growth model, by number of trajectory subgroup. Table S5. Fit indices for the trajectory model for difficulty initiating and maintaining sleep, by number of trajectory subgroup. Table S6. Model adequacy criteria for the trajectory model for difficulty initiating and maintaining sleep. Code S1. Example of SAS code for the calculation of entropy and mismatch. [file 12874_2022_1622_MOESM1_ESM.docx]

**Appendixes**

**Appendix 1: Simulation model**

We generated data for n=500 individuals with 5 repeated measures of the outcome $Y_{it}$, except for scenario 2 for which we considered a smaller dataset (n=300). Individual data $Y_{it}$were generated using the linear mixed model below, in which X~U(0,1) and $S_{0i}$ and $S_{1i}$are random effects that capture individual variation across the intercept and the linear component of the association between time and $Y_{it}$:

$$Y_{it}= {[\beta_{0}}^{j}{+{\beta_{1}}^{j}X_{i}+{{\beta_{2}}^{j}{X_{i}}^{2} +{\beta_{3}}^{j}{X_{i}}^{3}]+ [S}_{0i}}^{j}+{S_{1i}}^{j}X_{i}+ \varepsilon_{it}]$$

$\varepsilon_{it} \sim N(0, \sigma^{2})$, $X\sim U(0,1)$

$$S_{0i} \sim N(0, \sigma_{0i}^{2}), S_{1i} \sim N(0, \sigma_{1i}^{2}).$$

Individual sequences $Y_{it}$ were then indexed using a time variable ranging from 1 to 5. Hence, while individual patterns in the same subgroup shared a similar shape provided by the same values of the $\beta$ coefficients, within subgroup heterogeneity was introduced by both the error term ($\varepsilon_{it})$ and the random effects.

Table S1: Parameter values used in the simulation for each scenario

| Scenarios |  | Subgroup 1 | | | | | Subgroup 2 | | | | | Subgroup 3 | | | | |
| --- | --- | --- | --- | --- | --- | --- | --- | --- | --- | --- | --- | --- | --- | --- | --- | --- |
|  |  | t1 | t2 | t3 | t4 | t5 | t1 | t2 | t3 | t4 | t5 | t1 | t2 | t3 | t4 | t5 |
| Scenario 1: Three distinct trajectory subgroups | $\beta_{0}$ | 20 | 30 | 40 | 55 | 65 | 70 | 60 | 45 | 30 | 15 | 100 | 90 | 85 | 70 | 55 |
|  | $\beta_{1}$ | 15 | 15 | 15 | 15 | 15 | 5 | 5 | 5 | 5 | 5 | 5 | 5 | 5 | 5 | 5 |
|  | $\beta_{2}$ | 2 | 2 | 2 | 2 | 2 | 2 | 2 | 2 | 2 | 2 | 2 | 2 | 2 | 2 | 2 |
|  | $\beta_{3}$ | 0 | 0 | 0 | 0 | 0 | 0 | 0 | 0 | 0 | 0 | 0 | 0 | 0 | 0 | 0 |
|  | $\sigma^{2}$ | 100 | 100 | 100 | 100 | 100 | 100 | 100 | 100 | 100 | 100 | 100 | 100 | 100 | 100 | 100 |
|  | $\sigma_{0}^{2}$ | 24 | 24 | 24 | 24 | 24 | 24 | 24 | 24 | 24 | 24 | 24 | 24 | 24 | 24 | 24 |
| Scenario 2: Different range of outcome values across subgroups | $\beta_{0}$ | 20 | 30 | 40 | 55 | 65 | 70 | 60 | 45 | 30 | 15 | 200 | 35 | 15 | 10 | -100 |
|  | $\beta_{1}$ | 15 | 15 | 15 | 15 | 15 | 5 | 5 | 5 | 5 | 5 | 5 | 5 | 5 | 5 | 5 |
|  | $\beta_{2}$ | 2 | 2 | 2 | 2 | 2 | 2 | 2 | 2 | 2 | 2 | 2 | 2 | 2 | 2 | 2 |
|  | $\beta_{3}$ | 0 | 0 | 0 | 0 | 0 | 0 | 0 | 0 | 0 | 0 | 0 | 0 | 0 | 0 | 0 |
|  | $\sigma^{2}$ | 100 | 100 | 100 | 100 | 100 | 100 | 100 | 100 | 100 | 100 | 100 | 100 | 100 | 100 | 100 |
|  | $\sigma_{0}^{2}$ | 24 | 24 | 24 | 24 | 24 | 24 | 24 | 24 | 24 | 24 | 24 | 24 | 24 | 24 | 24 |
| Scenario 3: Time point-specific overlap in the distribution of the outcome | $\beta_{0}$ | 20 | 25 | 30 | 35 | 40 | 70 | 75 | 80 | 85 | 90 | 70 | 65 | 60 | 55 | 50 |
|  | $\beta_{1}$ | 15 | 15 | 15 | 15 | 15 | 15 | 15 | 15 | 15 | 15 | 15 | 15 | 15 | 15 | 15 |
|  | $\beta_{2}$ | 2 | 2 | 2 | 2 | 2 | 2 | 2 | 2 | 2 | 2 | 2 | 2 | 2 | 2 | 2 |
|  | $\beta_{3}$ | -0.5 | -0.5 | -0.5 | -0.5 | -0.5 | -0.5 | -0.5 | -0.5 | -0.5 | -0.5 | -0.5 | -0.5 | -0.5 | -0.5 | -0.5 |
|  | $\sigma^{2}$ | 100 | 100 | 100 | 100 | 100 | 100 | 100 | 100 | 100 | 100 | 49 | 49 | 49 | 49 | 49 |
|  | $\sigma_{0}^{2}$ | 24 | 24 | 24 | 24 | 24 | 24 | 24 | 24 | 24 | 24 | 64 | 64 | 64 | 64 | 64 |
| Scenario 4 Increasing within **s**ubgroup variance | $\beta_{0}$ | 20 | 25 | 30 | 35 | 40 | 50 | 55 | 60 | 65 | 70 | 80 | 85 | 90 | 95 | 100 |
|  | $\beta_{1}$ | 15 | 15 | 15 | 15 | 15 | 15 | 15 | 15 | 15 | 15 | 15 | 15 | 15 | 15 | 15 |
|  | $\beta_{2}$ | 2 | 2 | 2 | 2 | 2 | 2 | 2 | 2 | 2 | 2 | 2 | 2 | 2 | 2 | 2 |
|  | $\beta_{3}$ | 0 | 0 | 0 | 0 | 0 | 0 | 0 | 0 | 0 | 0 | 0 | 0 | 0 | 0 | 0 |
|  | $\sigma^{2}$ | 20 | 25 | 64 | 81 | 100 | 20 | 25 | 64 | 81 | 100 | 20 | 25 | 64 | 81 | 100 |
|  | $\sigma_{0}^{2}$ | 24 | 24 | 24 | 24 | 24 | 24 | 24 | 24 | 24 | 24 | 24 | 24 | 24 | 24 | 24 |
| Scenario 5: Rainbow | $\beta_{0}$ | 20 | 25 | 30 | 35 | 40 |  |  |  |  |  |  |  |  |  |  |
|  | $\beta_{1}$ | 15 | 15 | 15 | 15 | 15 |  |  |  |  |  |  |  |  |  |  |
|  | $\beta_{2}$ | 2 | 2 | 2 | 2 | 2 |  |  |  |  |  |  |  |  |  |  |
|  | $\beta_{3}$ | -0.5 | -0.5 | -0.5 | -0.5 | -0.5 |  |  |  |  |  |  |  |  |  |  |
|  | $\sigma^{2}$ | 100 | 100 | 100 | 100 | 100 |  |  |  |  |  |  |  |  |  |  |
|  | $\sigma_{0}^{2}$ | 24 | 24 | 24 | 24 | 24 |  |  |  |  |  |  |  |  |  |  |
| Scenario 6: No temporal patterns | $\beta_{0}$ | 25 | 25 | 27 | 27 | 28 |  |  |  |  |  |  |  |  |  |  |
|  | $\beta_{1}$ | 15 | 15 | 15 | 15 | 15 |  |  |  |  |  |  |  |  |  |  |
|  | $\beta_{2}$ | 2 | 2 | 2 | 2 | 2 |  |  |  |  |  |  |  |  |  |  |
|  | $\beta_{3}$ | 5 | 5 | 5 | 5 | 5 |  |  |  |  |  |  |  |  |  |  |
|  | $\sigma^{2}$ | 200 | 200 | 200 | 200 | 200 |  |  |  |  |  |  |  |  |  |  |
|  | $\sigma_{0}^{2}$ | 150 | 150 | 150 | 150 | 150 |  |  |  |  |  |  |  |  |  |  |
|  | $\sigma_{1}^{2}$ | 100 | 100 | 100 | 100 | 100 |  |  |  |  |  |  |  |  |  |  |
|  | $\sigma_{0}\sigma_{1}$ | 0 | 0 | 0 | 0 | 0 |  |  |  |  |  |  |  |  |  |  |

**Table S2:** Fit indices for the trajectory model using group-based trajectory modeling, by number of trajectory subgroup for scenarios 1-3

|  | Scenario 1: Three distinct trajectory subgroups | | | Scenario 2: Different range of outcome values across subgroups | | | Scenario 3: Time point-specific overlap in the distribution of the outcome | | |
| --- | --- | --- | --- | --- | --- | --- | --- | --- | --- |
| Models | BIC | Bayes factor | Smallest group size | BIC | Bayes factor | Smallest group size | BIC | Bayes factor | Smallest group size |
| 1-subgroup | -11575.93 |  |  | -8003.36 |  |  | -3184.06 |  |  |
| 2-subgroup | -10980.16 | 1191.54 | 167 | -7296.34 | 1414.02 | 100 | -3061.36 | 245.40 | 79 |
| 3-subgroup | -10314.23 | 1331.87 | 164 | -8067.20 | -1541.72 | 300 | -3036.01 | 50.69 | 20 |
| 4-subgroup | -10310.52 | 7.41 | 60 | -8078.17 | -21.94 | 300 | -3033.80 | 4.43 | 15 |
| 5-subgroup | -10252.98 | 115.09 | 72 | -8089.14 | -21.94 | 300 | -3043.88 | -20.16 | 15 |
| 6-subgroup | -10264.45 | -22.94 | 1 | -8100.11 | -21.94 | 300 | -3053.96 | -20.16 | 15 |
| 7-subgroup | -10256.81 | 15.28 | 9 | -8111.08 | -21.94 | 300 | -3062.04 | -16.14 | 4 |
| 8-subgroup | -10234.04 | 45.53 | 5 | -8122.05 | -21.94 | 300 | -3070.76 | -17.46 | 6 |
| 9-subgroup | -10281.37 | -94.65 | 1 | -7441.11 | 1361.88 | 100 | -3073.73 | -5.93 | 1 |
| 10-subgroup | -10267.06 | 28.61 | 5 | -8143.99 | -1405.76 | 300 | -3083.81 | -20.16 | 1 |

The part highlighted in grey corresponds to the number of selected trajectories. The latter is selected using the BIC approximation to the Bayes factor as long as the difference between the two adjacent models was greater than 10.

**Table S3:** Fit indices for the trajectory model using group-based trajectory modelling, by number of trajectory subgroup for scenarios 4-6

|  | Scenario 4: Increasing within subgroup variance | | | Scenario 5: Rainbow effect | | | Scenario 6: No temporal patterns | | |
| --- | --- | --- | --- | --- | --- | --- | --- | --- | --- |
| Models | BIC | Bayes factor | Smallest group size | BIC | Bayes factor | Smallest group size | BIC | Bayes factor | Smallest group size |
| 1-subgroup | -11755.25 |  |  | -9842.11 |  |  | -11098.45 |  |  |
| 2-subgroup | -10669.89 | 2170.71 | 246 | -9681.41 | 321.38 | 244 | -10796.12 | 604.65 | 175 |
| 3-subgroup | -9900.63 | 1538.51 | 165 | -9659.00 | 44.82 | 82 | -10716.63 | 158.99 | 48 |
| 4-subgroup | -9850.03 | 101.21 | 84 | -9670.74 | -23.47 | 82 | -10685.78 | 61.68 | 27 |
| 5-subgroup | -9799.71 | 100.65 | 66 | -9676.05 | -10.62 | 46 | -10686.24 | -0.92 | 12 |
| 6-subgroup | -9750.19 | 99.03 | 69 | -9687.58 | -23.06 | 46 | -10693.42 | -14.36 | 7 |
| 7-subgroup | -9749.74 | 0.90 | 24 | -9699.31 | -23.47 | 46 | -10708.58 | -30.31 | 6 |
| 8-subgroup | -9758.48 | -17.47 | 3 | -9711.05 | -23.47 | 46 | -10718.55 | -19.95 | 7 |
| 9-subgroup | -9764.17 | -11.38 | 3 | -9722.12 | -22.14 | 3 | -10722.62 | -8.13 | 4 |
| 10-subgroup | -9774.42 | -20.51 | 1 | -9733.67 | -23.10 | 1 | -10734.35 | -23.47 | 4 |

The part highlighted in grey corresponds to the number of selected trajectories. The latter is selected using the BIC approximation to the Bayes factor as long as the difference between the two adjacent models was greater than 10.

**Table S4:** Bayesian Information Criteria for the trajectory model using latent class growth model, by number of trajectory subgroup

| Models | Scenario 1: Three distinct trajectory subgroups | Scenario 2: Different range of outcome values across subgroups | Scenario 3: Time point-specific overlap in the distribution of the outcome | Scenario 4: Increasing within subgroup variance | Scenario 5: Rainbow effect | Scenario 6: No temporal patterns |
| --- | --- | --- | --- | --- | --- | --- |
| 1-subgroup | 22775.94 | 16004.37 | 20937.38 | 19578.64 | 19283.45 | 21304.37 |
| 2-subgroup | 20456.38 | 13361.23 | 20795.15 | 19424.45 | 19316.51 | 21327.06 |
| 3-subgroup | 20357.18 | 12892.41 | 20081.79 | 19455.01 | 19341.91 | 21351.28 |
| 4-subgroup | 20530.96 | 13429.68 | 20119.08 | 19400.88 | 19388.28 | 21394.21 |
| 5-subgroup | 20568.24 | 12333.96 | 20156.37 | 19438.17 | 19421.86 | 21431.50 |
| 6-subgroup | 20605.53 | 13497.96 | 20193.66 | 19552.31 | 19465.66 | 21468.79 |
| 7-subgroup | 20642.82 | 12402.41 | 20230.95 | 19593.03 | 19500.15 | 21506.08 |
| 8-subgroup | 20680.11 | 13566.57 | 20268.23 | 19626.89 | 19540.24 | 21527.79 |
| 9-subgroup | 20704.61 | 12470.85 | 20305.52 | 19664.18 | 19571.16 | 21596.62 |
| 10-subgroup | 20754.68 | 13634.85 | 21093.46 | 19722.76 | 19614.81 | 21618.13 |

**Table S5:** Fit indices for the trajectory model for difficulty initiating and maintaining sleep, by number of trajectory subgroup

| Models | BIC | Bayes factor | Smallest group size |
| --- | --- | --- | --- |
| 1-subgroup | -3719.05 |  |  |
| 2-subgroup | -3382.25 | 673.60 | 59 |
| 3-subgroup | -3297.18 | 170.14 | 47 |
| 4-subgroup | -3300.85 | -7.34 | 6 |
| 5-subgroup | -3266.41 | 68.89 | 7 |
| 6-subgroup | -3255.87 | 21.07 | 12 |
| 7-subgroup | -3260.30 | -8.85 | 12 |
| 8-subgroup | -3268.78 | -16.96 | 8 |
| 9-subgroup | -3282.55 | -27.54 | 5 |
| 10-subgroup | -3290.09 | -15.08 | 3 |

**Table S6:** Model adequacy criteria for the trajectory model for difficulty initiating and maintaining sleep

|  |  | Subgroups | | |
| --- | --- | --- | --- | --- |
| Criteria^a^ |  | 1 | 2 | 3 |
| Average posterior probability |  | 0.91 | 0.92 | 0.90 |
| Mismatch |  | **-2.02** | **2.53** | -0.51 |
| Relative entropy | 0.82 |  |  |  |

^a^APP > 0.70 and mismatch close to 0 suggest that the classification is good. Entropy close to 1 indicates that subjects were classified with more confidence. Bold values indicate poor classification.

**Code S1:** Example of SAS code for the calculation of entropy and mismatch

/******************************************

N: N total of observations

k: number of groups

Example for scenario 1 (3 distinct trajectory groups)

*******************************************/

**%MACRO** ***fit***;

%let N=500;

%let k=3;

data data.of3;

set data.of3;

IF grp1prb = **0** THEN pp1 = **0**; ELSE pp1 = grp1prb*log(grp1prb);

IF grp2prb = **0** THEN pp2 = **0**; ELSE pp2 = grp2prb*log(grp2prb);

IF grp3prb = **0** THEN pp3 = **0**; ELSE pp3 = grp3prb*log(grp3prb);

pp_j = pp1+pp2+pp3;

run;

PROC MEANS DATA=data.of3 NOPRINT;

VAR pp_j;

OUTPUT SUM=pp_sum OUT=of3_entropy;

RUN;

DATA of3_entropy;

SET of3_entropy;

DROP _TYPE_ _FREQ_;

entropy = -**1***pp_sum;

relative_entropy=**1** - (entropy/(&N*log(&k)));

RUN;

data os3;

set data.os3;

retain group;

if _n_=**1** then do;

group=**0**;

end;

group+**1**;

run;

proc freq data=data.of3;

table group/out=of3_count;

run;

data os3;

merge of3_count (keep=group count) os3;

by group;

run;

data os3;

set os3;

mismatch=PI-(count/&N***100**);

run;

proc print data=of3_entropy;

var relative_entropy;

run;

proc print data=os3;

var group mismatch;

run;

**%MEND**;

%***fit***;
